# Analysis of Structural Parameters of Steel–NC–UHPC Composite Beams

**DOI:** 10.3390/ma16165586

**Published:** 2023-08-11

**Authors:** Dawei Zhang, Xiaogang Ma, Huijie Shen, Songsong Guo, Chao Liu

**Affiliations:** 1 Shanghai Pudong Architectural Design & Research Institute Co., Ltd., Shanghai 201204, China; 2 College of Civil Engineering, Tongji University, Shanghai 200092, China

**Keywords:** steel–normal concrete (NC)–Ultra High Performance Concrete (UHPC) composite slabs, interface tests, design, parametric analysis

## Abstract

The cracking of the negative moment area of steel–normal concrete (NC) composite bridges is common owning to the low tensile strength of concrete. In order to solve the problem, Ultra High Performance Concrete (UHPC) is used to enhance the tensile performance of the negative moment area. This paper conducted interface experiments to study the bonding behaviour of the UHPC–NC interface. The design parametric analysis of steel–NC–UHPC composite bridges was carried out based on the interface experimental results. Firstly, slant shear tests and flexural shear tests were carried out to study the rationality of the interface handling methods. Then, the finite element model was used to analyze the state of every component in the composite beams based on experimental results, such as the stress of UHPC, concrete and steel plate. Finally, the calculation results of finite analysis were compared and summarized. It is concluded that (1) the chiseling interface can meet the utilization requirements of physical bridges. The average shear stress and flexural tensile strength of the chiseling interface are 10.29 MPa and 1.93 MPa, respectively. In the failure state, a slight interface damage occurs for specimens with a chiseling interface. (2) The influence on overall performance is different for changes in different design parameters. The thickness of concrete has a significant influence on the stress distribution of composite slabs. (3) Reliable interface simulation is conducted in the finite element models based on interface test results. The stress variation patterns are reflected in the change of design parameters.

## 1. Introduction

Steel concrete composite beams achieve higher strength and stiffness than concrete beams. The tensile strength of steel and compressive strength of concrete are fully utilized in steel–concrete composite bridges [1]. However, there are still many problems occurring in steel concrete composite beams with the increase in the span of bridges, especially in the negative moment area near the support. The cracking of bridge slabs is common and has a negative influence on the normal service of bridges [2]. In order to enhance the tensile performance of the negative moment area, many methods are proposed, such as prestressing force, changing the construction process, and using new materials. Considering the thickness of composite slabs, prestressing force is difficult and has a bad effect on the construction. As for changing the construction process, it is easy to conduct, such as using a conversion system in the construction. However, the enhancing effects of this method are slight. Currently, using a new material which has higher tensile strength to replace concrete is popular in practical engineering.

UHPC is a material with higher tensile strength and good ductility [3,4]. UHPC was first used by the U.S. Army Corps of Engineers in the late 1980s and became commercially available in the 2000s [5]. The first application of UHPC in bridge construction was in a pedestrian bridge in Canada in 1997 [6]. Since then, UHPC has been used for various applications, such as highway infrastructure, buildings, marine structures, and blast-resistant structures [7].

The development process of UHPC involves industrial by-products, such as slag and silica fume instead of Portland cement [8], adding steel fibers for reinforcement [9], and applying heat or steam curing to enhance strength and durability [10]. With the maturity of design and construction of UHPC, the cost has decreased to some degree [11]. This is mainly due to the use of local materials and the decrease in fiber volume ratio. It was reported that the cost of UHPC was reduced by 40% when local materials were used and the fiber content was reduced from 2% to 1%. This promoted the development and further application of UHPC [12]. UHPC has been used in bridge slabs for various purposes, such as accelerated construction [13], long-span bridge design, repair and strengthening of existing bridges [14], and improved durability and resistance to corrosion [15]. UHPC can also reduce the self-weight and thickness of bridge slabs compared to conventional concrete slabs.

Besides, reliable interface between UHPC and concrete is crucial to achieve the shared force. The interface bond strength is influenced by various factors, such as surface treatment [16,17], curing conditions [18], roughness and substrate strength [19], loading modes and environmental conditions [20]. Different experimental methods, such as flexural tensile tests, slant shear tests, direct shear tests and splitting tensile tests, can be used to evaluate the interface bond strength and durability.

This paper studies the bonding behaviour of UHPC-NC interface, and conducts a parametric analysis of the design parameters of steel–NC–UHPC composite beams. Firstly, slant shear tests and flexural tensile tests were carried out to study the interface performance with different handling methods. Then, a finite model was used to analyze the state of every component in steel–NC–UHPC composite beams. Finally, the calculation results of finite analysis were compared and summarized. This study provides support and reference for the design and construction of steel–NC–UHPC composite slabs.

## 2. The Bonding Performance of the Interface

### 2.1. Materials

The concrete used in the present research was obtained from an original concrete, produced and delivered by a ready-mix concrete company. After 28 days of curing conditions, an average compressive strength of 43.27 MPa was obtained for concrete. The Ultra High-Performance Concrete (UHPC) was cast in place and cured according to the construction environment. After 28 days, an average compressive strength of 122.84 MPa was obtained for UHPC. The constituents of concrete are listed in Table 1.

UHPC was designed according to the requirements of tensile strength and shrinkage creep deformation. The components of UHPC are listed in Table 2.

The characteristics of steel fibers in the UHPC can be seen in Table 3.

### 2.2. Interface Tests

#### 2.2.1. Slant Shear Tests

##### Specimen Design

**Figure 1:**
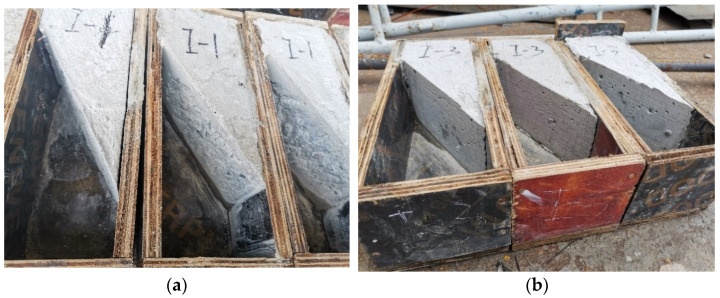
The slant shear specimens before pouring UHPC. (**a**) Chiselling surface; (**b**) Smooth surface.

Six prismatic specimens with 100 × 100 × 300 mm^3^, grouped in 2 sets of 3 specimens, were produced, with the interface surface at 30° with the vertical. Each of these was composed of half in NC and half in UHPC, 2 sets corresponding to the 2 types of surface handling methods considered, respectively. The interface processing methods included chiseling and smooth interface. In addition, the interface was always dried before casting the UHPC overlay. The slant shear specimens are shown in Figure 1.

##### Fabrication

Two types of surface processing methods were considered: smooth surface and chiseling surface. Surface laitance is only cleaned in the smooth surface. The chiseling surface shows the coarse aggregate, and the valley peak is about 15 mm.

##### Load

**Figure 2:**
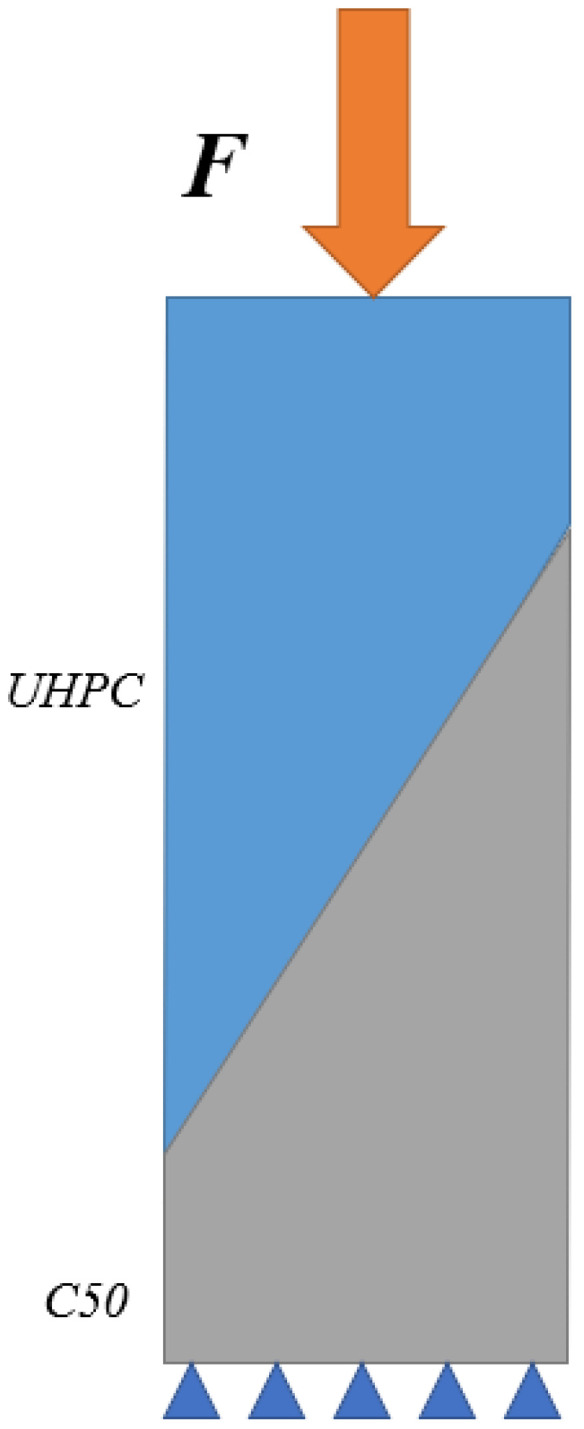
Loading diagram of slant shear tests.

The slant shear tests were conducted in the universal testing machine. The load was recorded via a force transducer. Before loading, the loading area should be cleaned and kept level, which ensures uniform loading. The loading diagram of slant shear tests is shown in Figure 2.

##### Failure Type

**Figure 3:**
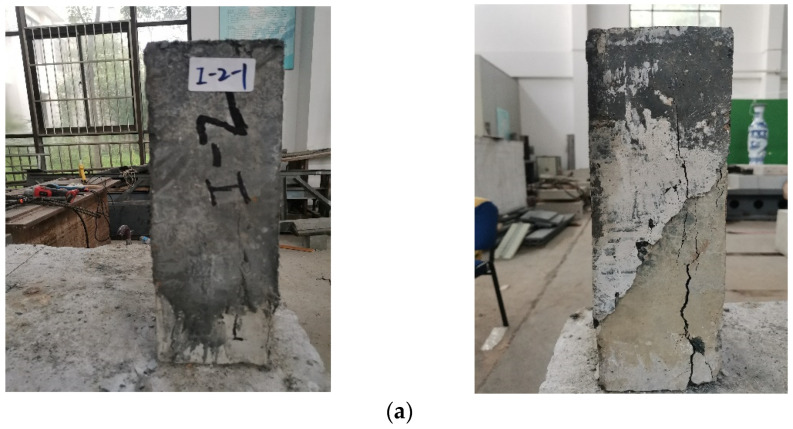

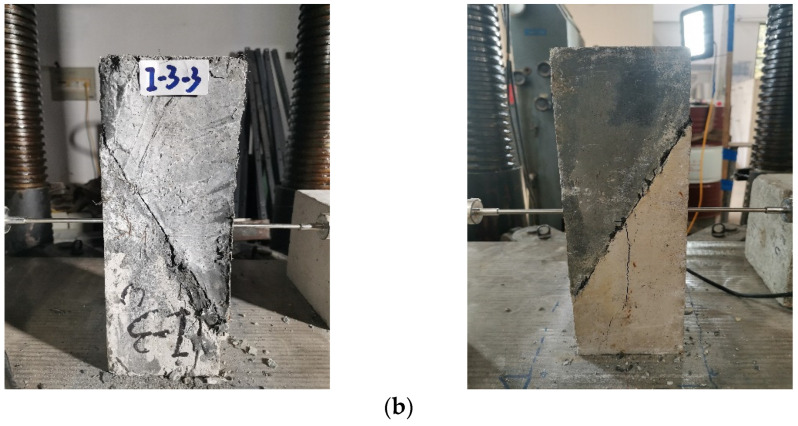
Failure state of slant shear specimens. (**a**) Slant specimens with chiselling interface; (**b**) Slant specimens with smooth interface.

The phenomenon of slant specimens with different interfaces is diverse. From Figure 3, it can be seen that there was a slight slip for the chiseling interface. In the meantime, the bottom concrete was broken. For smooth surface, the failure state is different to that of the first case. The interface was broken and there was no apparent damage in the concrete and UHPC. It reflects a brittle failure with sudden interface slip. Thus, smooth surface should not be used in practical engineering.

As for slant shear tests, there are three failure types of interface in total.

(1) A: interface of concrete and UHPC is invalid, concrete is intact;
(2) B: interface of concrete and UHPC is invalid, there is a slight damage to concrete;
(3) C: interface of concrete and UHPC has slight damage, concrete is crushed.

##### Slant Shear Strength

**Figure 4:**
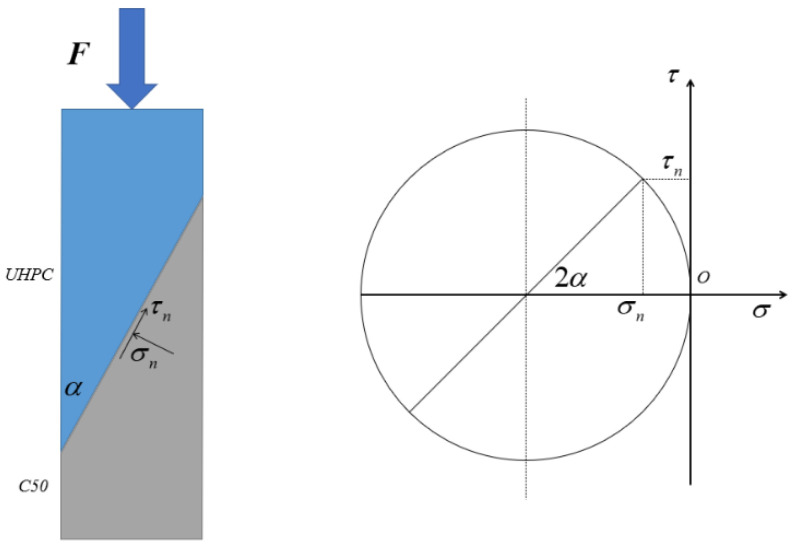
Shear stress calculation and Mohr-Coulomb circle.

The normal shear stress of interface can be given according to the requirements of ACI 546 [21]. The diagram of shear stress calculation and Mohr-Coulomb circle is shown in Figure 4.

(1) $$\tau =\frac{F}{A}$$

Normal stress of interface can be calculated with Equation (2).

(2) $$\sigma =\frac{P{\left(\sin\alpha \right)}^{2}}{A}$$

Shear stress of interface can be calculated with Equation (3).

(3) $$\tau =\frac{0.5P\sin(2\alpha )}{A}$$

The slant shear strength of interface should be calculated according to the bonding area. The interface shear strength is shown in Table 4.

From Figure 3 and Table 4, it can be seen that the shear stress and normal stress of smooth interface are low and the failure type of specimens with smooth surface reflects interface shear failure. Smooth surface cannot meet the needs of interface roughness. Besides, there is a slight slip of the interface and the bottom concrete is broken for specimens with a chiseled interface. The failure of concrete is prior to the occurrence of interface, which meets the actual requirements.

#### 2.2.2. Flexural Tensile Tests

##### Specimen Design

**Figure 5:**
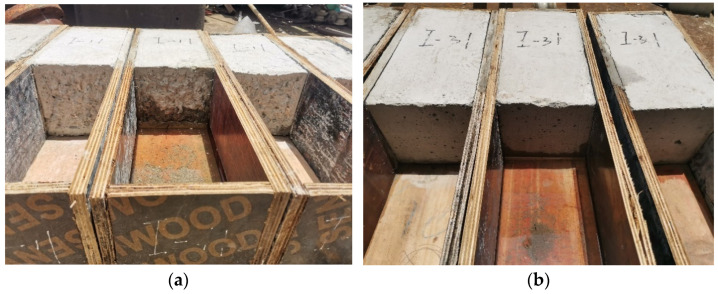
The flexural tensile specimens. (**a**) Chiselling surface; (**b**) Smooth surface.

Six prismatic specimens with 150 × 150 × 600 mm^3^, grouped in 2 sets of 3 specimens, were produced with the interface surface at 90° with the vertical. Each of these was composed of half in NC and half in UHPC, 2 sets corresponding to the 2 types of surface handling methods considered. The specimen before pouring UHPC is shown in Figure 5.

##### Load

**Figure 6:**
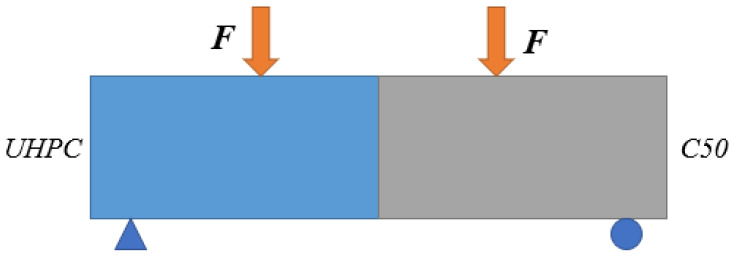
Loading diagram of flexural tensile tests.

The flexural tensile tests were conducted according to the Standard for test methods of concrete physical and mechanical properties (GB/T 50081—2019) [22]. The loading speed adopted was 0.05–0.08 MPa/s. The loading would stop when the specimens were broken. The loading diagram of flexural tensile tests is shown in Figure 6.

##### Failure Type

The failure of flexural tensile tests can be divided into three types according to the test phenomenon.
NumberFailure typeIflexural tensile failure of UHPC sideIIflexural tensile failure of interfaceIIIflexural tensile failure of concrete side

**Figure 7:**
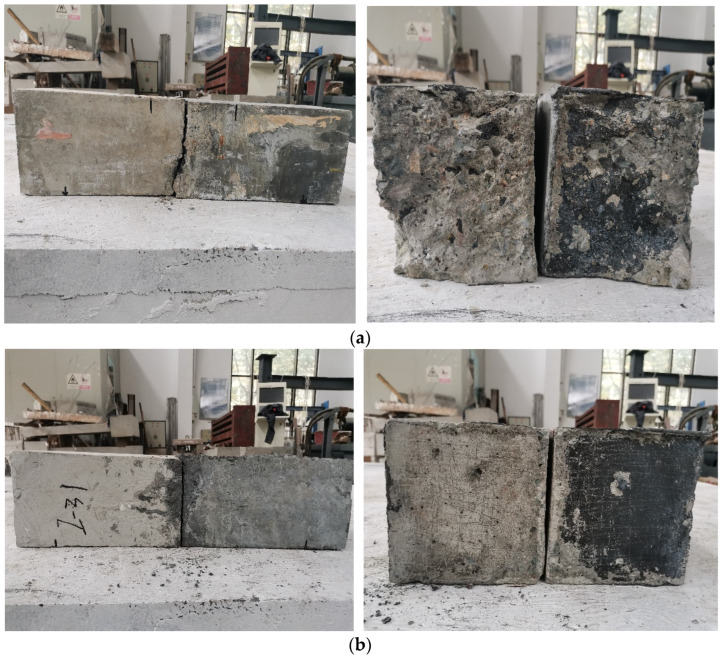
The phenomenon of flexural tensile tests. (**a**) Flexural tensile specimens with chiseling surface; (**b**) Flexural tensile specimens with smooth surface.

The failure types of flexural tensile specimens can be seen in Figure 7. The failure mode of chiseling specimen manifests as flexural tensile failure on the concrete side. In the meantime, there are many aggregates of concrete which can be attached to the UHPC section. For specimens with a smooth surface, interface failure occurs. There is no apparent damage to the concrete and UHPC.

##### Flexural Tensile Strength

The flexural tensile strength can be calculated by Equations (4)–(6).

(4) $$\sigma =\frac{M}{W}$$

(5) $$M=\frac{FL}{6}$$

(6) $$W=\frac{bh^{2}}{6}$$

Thus,

(7) $$\sigma =\frac{FL}{bh^{2}}$$

where, *σ* is the flexural tensile stress of the interface; *M* is the bending moment in the interface; *W* is the resistance moments of the section; *F* is the concentrated load; *L* is the effective span of flexural tensile specimens; *b* is the width of the section; *h* is the height of specimens.

From Figure 7 and Table 5, we can see that there are apparent differences in adhesive capacity for specimens with different interfaces. For specimens with smooth surface, the adhesive capacity is the lowest and the failure position is in the interface. There is no damage to the concrete and UHPC. It should not be adopted in the design. For specimens with a chiseling surface, the destruction phenomenon is evident. There is a slight damage on the concrete side with the failure of interface.

## 3. Finite Model

### 3.1. Specimen Design

The bridge is a three-span continuous steel–concrete composite girder bridge. The span is 40 + 60 + 40 = 140 m, and the width is 16.5 m. The layout of the bridge slab is: 0.5 m (anti-collision guardrail) +7.5 m (motor vehicle lane) +0.5 m (central separation pier) +7.5 m (motor vehicle lane) + 0.5 m (anti-collision guardrail). The bridge pavement is made with a 2 mm waterproof layer and 100 mm asphalt mix.

**Figure 8:**
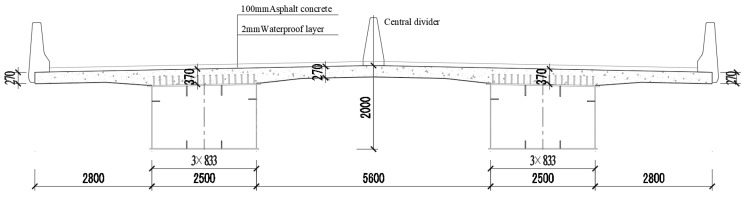
Cross section of composite beams (unit: mm).

The bridge deck is a composite bridge slab, in which the inner thickness is 270 mm, and the thickness of the root of the cantilever slab is 370 mm. The thickness of the bottom steel plate is 8 mm. The steel plate and concrete are connected with T-type steel ribs and studs. The cross section of steel–NC–UHPC composite beam is shown in Figure 8.

### 3.2. Element Election

Shell181 element is used to simulate the steel beam, and the solid185 element is used to simulate concrete and UHPC using the Ansys software 2022 R1 [23]. Reinforcement bars and internal steel ribs are connected to the concrete and UHPC layer by coupling elements. Constraints are used to constrain the lower nodes of the main girder according to the constraints of the three-span continuous beam.

**Figure 9:**
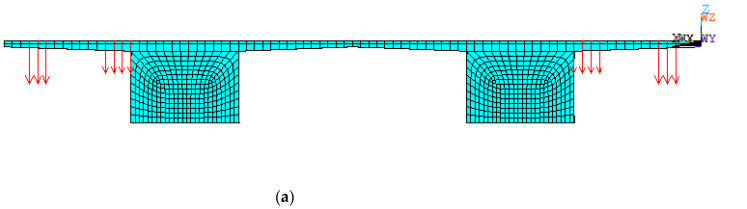

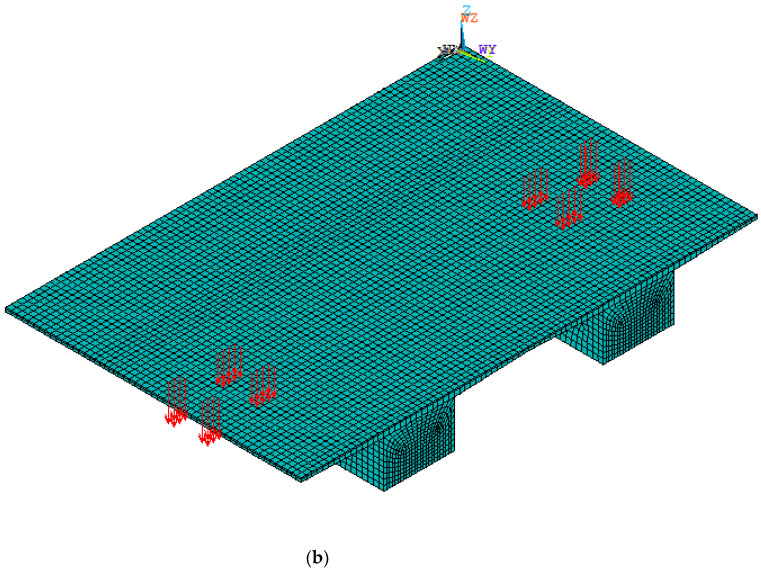
Finite model of composite bridges. (**a**) Front view; (**b**) Side view.

The finite model contains a total of 35,586 nodes and 32,220 elements. The model is constrained, with one fixed end and one simply supported end, and the loads are arranged according to lanes for vehicle loads. The finite model is shown in Figure 9.

### 3.3. Materials

The proprieties of concrete and UHPC are the same as these of the interface tests. Elastic modulus of concrete and UHPC are 30,041 MPa and 41,617 MPa, respectively. Poisson’s Ratio is 0.2. The yield strength and tensile strength of steel bars, steel ribs and steel plate are adopted according to the related guidelines. All the reinforcements had the same elastic modulus of 200 GPa and Poisson’s Ratio of 0.3. The elastic modulus and Poisson’s Ratio of studs and steel ribs were the same as those of reinforcements. All components are in the elastic state in the finite analysis.

### 3.4. Interface

The common nodes are used to simulate the behaviour of steel plate and concrete, because studs and steel ribs provide enough interface shear capacity. From the results of the interface tests of UHPC and concrete, it is indicated that the shear capacity of the chiseling interface meets the requirements of related guidelines. Thus, the interface slip is neglected in the current finite model. The steel bars and steel ribs are coupled with the concrete and UHPC layer.

### 3.5. Load

**Figure 10:**
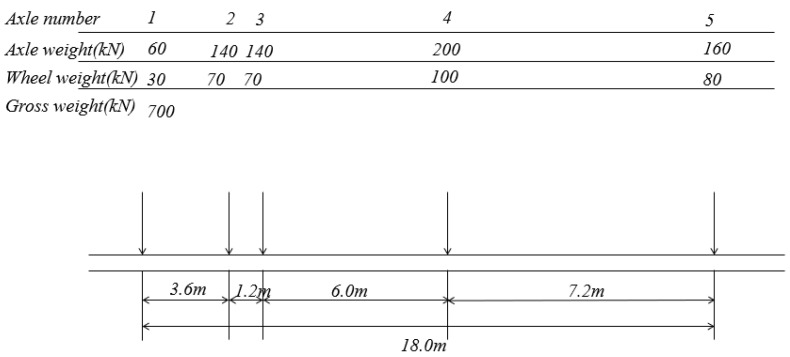
Arrangement of vehicle loading.

In order to ensure that the design of the composite bridge deck meets the requirements of codes under vehicle loading, a local finite model is established for finite element calculation and analysis. In accordance with the existing design of composite slabs, the spacing of horizontal partitions is 3.5 m and three sections are selected to analyze the behaviour. The vehicle loading adopts axial loading with a load of 140 kN. The elevation arrangement of vehicle loading is shown in Figure 10.

## 4. Parametric Analysis

Variable analysis is carried out to understand the sensitivity and distribution of the stresses of different parts with change in the design parameters of composite beams. This provides a reference for the analysis of the main factors in the design of composite beams. In the parametric analysis, the thickness of concrete changes from 0.08 m to 0.3 m with a 0.05 m-thick UHPC layer. The parameter can be seen in Table 6. The effects of the thickness of steel plate are also compared and considered. The thickness of steel plate changes from 0.008 m to 0.02 m, as shown in Table 7.

### 4.1. Concrete

**Figure 11:**
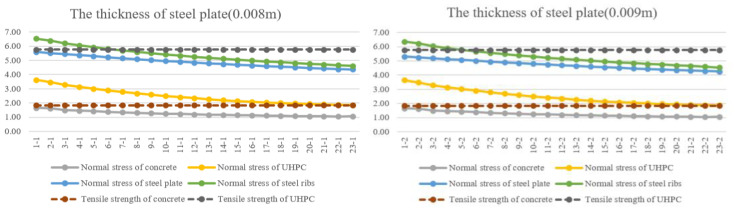

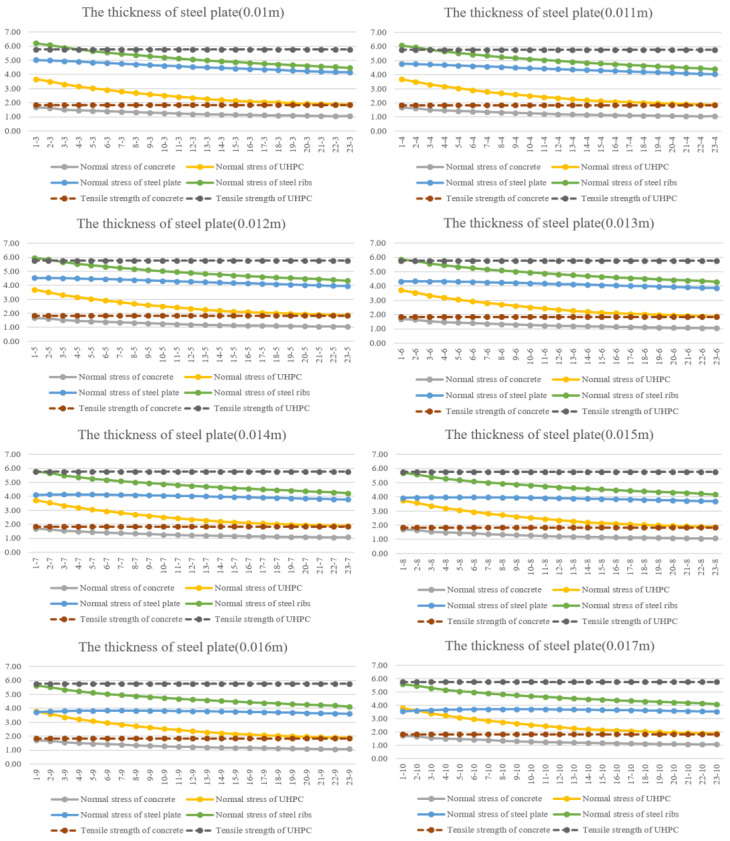

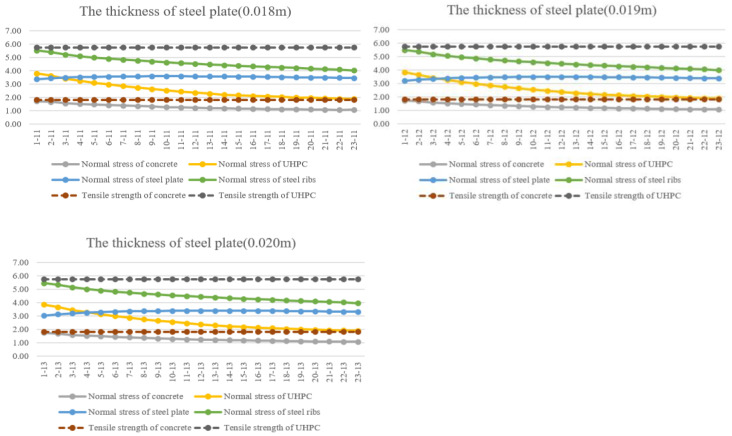
The stress of every component in composite beams-1. (In the picture, 1-1 indicates number-concrete-1 and number-steel plate-1).

As we all know, thickness has a great effect on the bearing capacity of bridge slabs. In the meantime, increase in thickness of slabs will enhance mechanical performance in the service state. In order to reveal the influence on normal stress of every component with the change in thickness of a concrete layer, a series of finite models are modelled and calculated. The normal stress of concrete, UHPC, steel plate and steel ribs is extracted from the results, as in Figure 11.

As can be seen from Figure 11, the normal stress of concrete, UHPC, steel plate and steel ribs gradually decreases with the increase of thickness of concrete. Although there is a similar trend, the magnitude of change of stress is not completely the same. The change in normal stress of the UHPC layer and steel ribs is relatively more evident than that of steel plate and concrete. In the meantime, the normal stress of concrete and UHPC are lower than the design tensile strength, which meets the requirements of engineering.

### 4.2. Steel Plate

**Figure 12:**
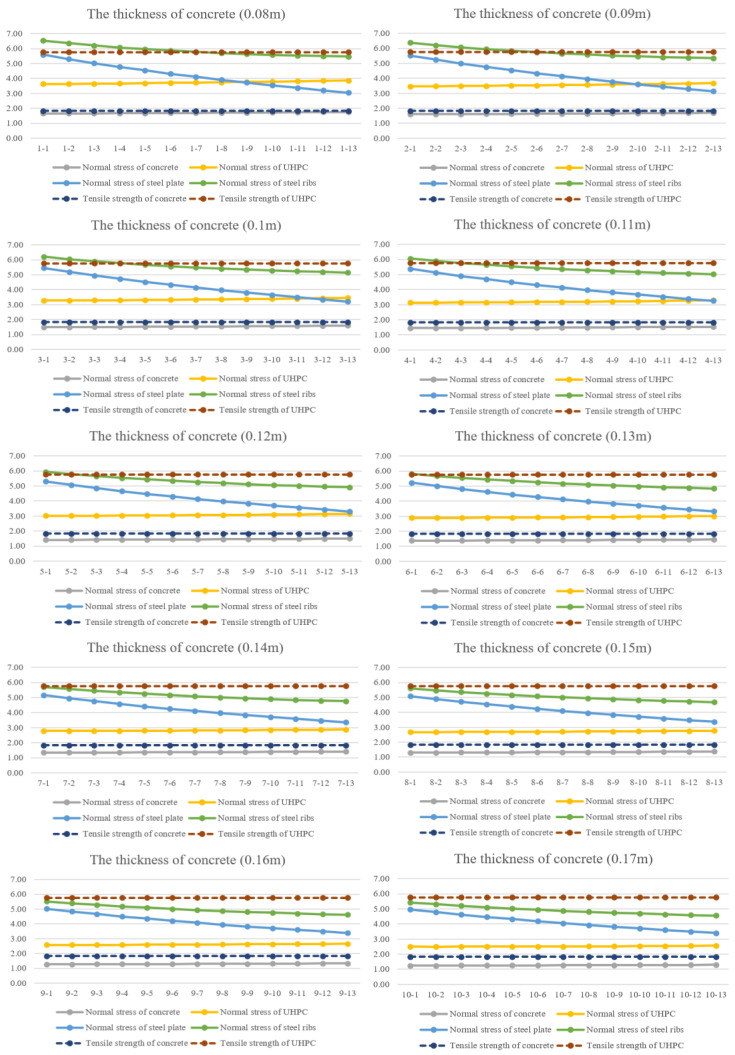

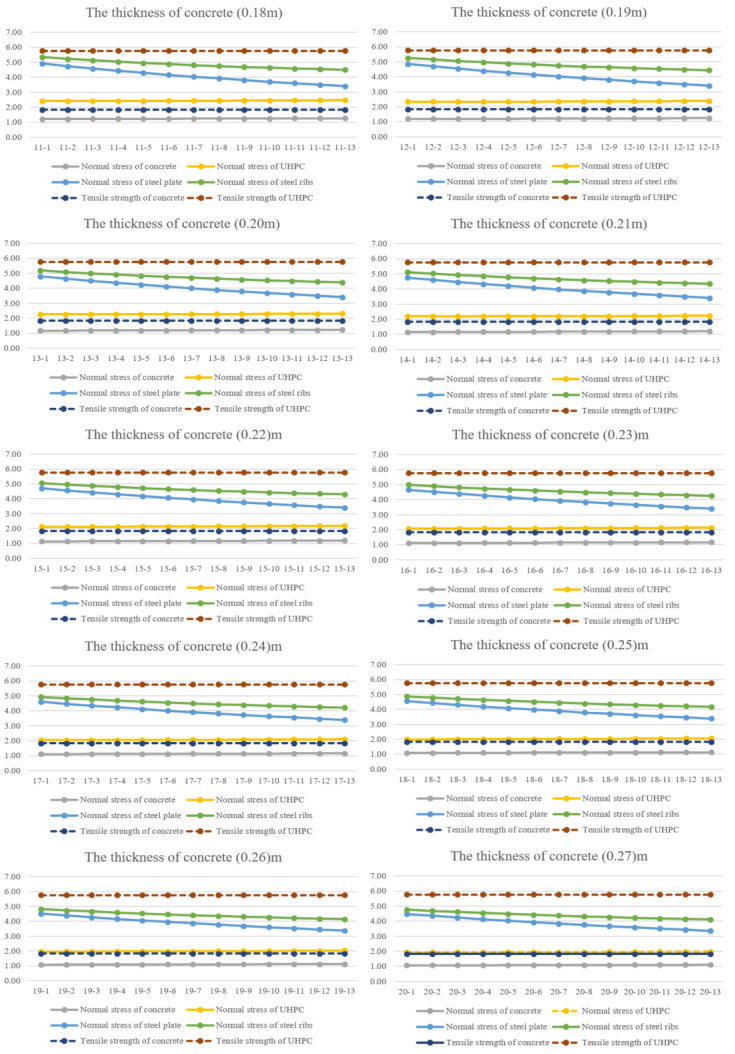

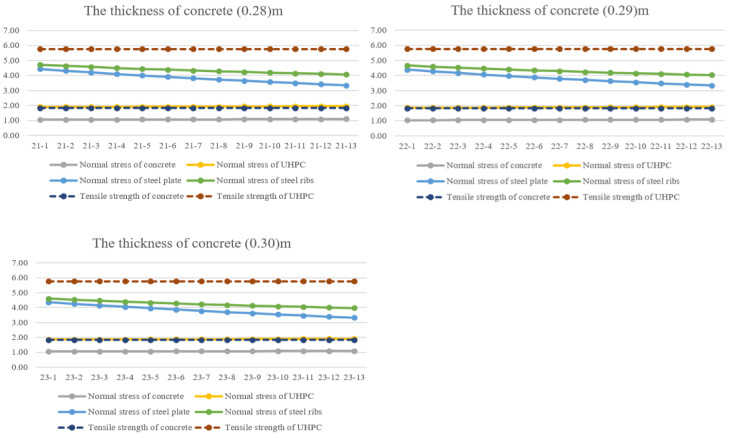
The stress of every component in composite beams-2. (In the picture, 1-1 indicates number-steel plate-1 and number-concrete-1).

For composite slabs with a large cantilever span, bottom steel plate not only provides construction templates, but also improves the mechanical performance of bridge slabs, with higher compressive and tensile strength than that of concrete. In order to show the influence on normal stress of every component with change in the thickness of steel plate, a series of finite models is modelled and calculated. The normal stress of concrete, UHPC, steel plate and steel ribs is extracted from the results, as seen in Figure 12.

It is shown in Figure 12 that the change is similar to that in Figure 11. With the same thickness of concrete and UHPC layer, the normal stress of concrete, UHPC, steel plate and steel ribs gradually decreases with the increase in thickness of steel plate. The change in normal stress of steel plate and steel ribs is relatively more apparent than that of steel plate and concrete.

### 4.3. The Interval of Steel Ribs

In conjunction with the actual construction process of the bridge, the steel plates initially function as a formwork to carry the self-weight of the concrete and UHPC. For this state of construction, the analysis of the stresses on the steel plates and steel ribs is carried out to determine a reasonable spacing for the steel ribs.

**Figure 13:**
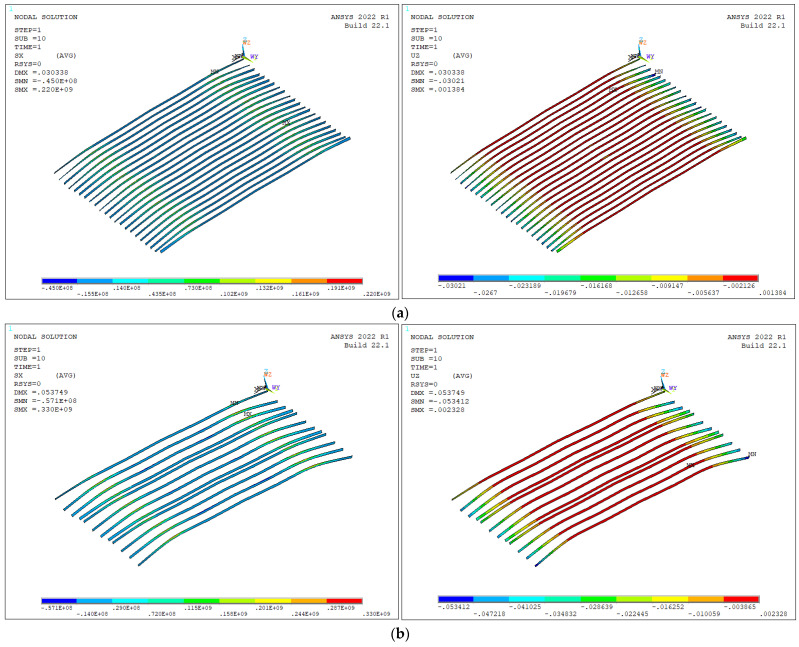
Stress and displacement of steel ribs. (**a**) Stress and displacement when the interval of steel ribs is 0.5 m; (**b**) Stress and displacement when the interval of steel ribs is 1 m.

According to the calculation results in Figure 13, the normal stress of the steel ribs gradually increases with the increase in the spacing under constant height of the steel ribs. The changes in stress and displacement are apparent. From Figure 13a,b, it is seen that the maximum displacement of steel ribs increases from 0.030 m to 0.053 m. The steel ribs prohibit the increase of the deformation and stress during construction phase. The smaller the spacing of the reinforcing ribs, the more effective.

## 5. Conclusions and Suggestions

In the paper, interface tests and finite analysis are used to study the interface shear behaviour and the flexural performance of steel–NC–UHPC composite slabs. The study reveals the mechanical performance of interface and composite slabs. The following conclusions are drawn:

(1) Chiseling interface prohibits the occurrence of interface failure and can be applied in the engineering. There is only slight interface damage to the chiseling surface based on the results of slant shear tests and flexural shear tests, which meets the needs of physical engineering.
(2) The phenomenon in the slant shear tests and flexural tensile tests is similar, which can reflect the interface bonding performance. Compared with direct shear tests and pull-out tests, it is easy to conduct slant shear tests and flexural tensile tests.
(3) The thickness of concrete, steel plate has a huge influence on the normal use of composite slabs. The normal stress of UHPC, concrete and steel plate will decrease with the increase in the thickness of concrete and steel plate. Besides, reducing the interval of steel ribs can effectively improve the flexural performance and decrease the stress of UHPC, concrete and steel plate.

There is a trend for UHPC to be used in bridge engineering in the future. The study of interface performance and parametric analysis of steel–NC–UHPC composite slabs provides reference and support for the design of composite bridges.

## Figures and Tables

**Table 1:** The components of concrete.

Materials	Cement	Mineral Powder	Fly Ash	Water	Sand	Rock	Admixture
Specification/Grade/Model	P.O42.5	S95	CII	/	medium sand	5–25 mm	ZWL-A-1X
mix ratio	1	0.2	0.09	0.45	1.69	2.88	0.013

**Table 2:** The components of UHPC.

Materials	Water (kg)	Premix (kg)	Steel Fiber (kg)	Admixture (kg)
UHPC/m^3^	177.3	2221.65	200	19.5

**Table 3:** The characteristics of steel fibers.

Number	Type	Value
1	Tensile strength (MPa)	2967
2	Length (mm)	12.99
3	Equivalent diameter (mm)	0.2
4	Length/equivalent diameter (mm)	65

**Table 4:** Interface shear strength.

Number	Interface Handling	Peak Load (kN)	Nominal Shear Stress (MPa)	Shear Stress (MPa)	Normal Stress (MPa)	Failure Type
I-1-1	Chiselling	212.00	10.60	9.18	5.30	C
I-1-2		269.34	13.47	11.66	6.73	C
I-1-3		231.83	11.59	10.04	5.80	C
I-2-1	Smooth	151.72	7.59	6.57	3.79	B
I-2-2		159.00	7.95	6.88	3.98	B
I-2-3		170.00	8.50	7.36	4.25	B

**Table 5:** Flexural shear strength.

Number	Interface Handling	Peak Load (kN)	Average Flexural Tensile Strength (MPa)	Failure Type
I-11-1	Chiselling	14.12	1.93	III
I-11-2		12.82		III
I-11-3		16.50		III
I-21-1	Smooth	12.00	1.58	II
I-21-2		12.20		II
I-21-2		11.10		II

**Table 6:** Thickness of concrete in the finite model.

Number-Concrete	Thickness of Concrete (m)	Thickness of UHPC Layer (m)
1	0.08	0.05
2	0.09	0.05
3	0.1	0.05
4	0.11	0.05
5	0.12	0.05
6	0.13	0.05
7	0.14	0.05
8	0.15	0.05
9	0.16	0.05
10	0.17	0.05
11	0.18	0.05
12	0.19	0.05
13	0.2	0.05
14	0.21	0.05
15	0.22	0.05
16	0.23	0.05
17	0.24	0.05
18	0.25	0.05
19	0.26	0.05
20	0.27	0.05
21	0.28	0.05
22	0.29	0.05
23	0.3	0.05

**Table 7:** Thickness of steel plate in the finite model.

Number-Steel Plate	Thickness of Steel Plate (m)
1	0.008
2	0.009
3	0.01
4	0.011
5	0.012
6	0.013
7	0.014
8	0.015
9	0.016
10	0.017
11	0.018
12	0.019
13	0.02

## Data Availability

The data used to support the findings of the study are included in the article.
